# Aberrant expression of UBE2C in endometrial cancer and its correlation to epithelial mesenchymal transition

**DOI:** 10.1097/MD.0000000000033834

**Published:** 2023-05-17

**Authors:** Yan Zhang, Xueting Li, Yingying Gong, Danli Du, Huilei Chen, Lei Liu, Zenong Cheng

**Affiliations:** a Department of Gynaecology and Obstetrics, Bengbu City, China; b Department of Pathology, the First Affiliated Hospital of Bengbu Medical University, Bengbu City, China; c Department of Pathology, Bengbu Medical University, Bengbu City, China.

**Keywords:** endometrial cancer, epithelial-mesenchymal transition, UBE2C, WNT5α, ZEB1

## Abstract

Ubiquitin-conjugating enzyme E2C (UBE2C), its overexpression promotes tumor progression, is a key component of the ubiquitin conjugating proteasome complex. Epithelial-mesenchymal transition, which is lost epithelial features and gained mesenchymal features in some epithelial cancers, is involved in epithelial cancers’ invasiveness and metastasis. The aim of this study is to detect the expression of UBE2C, WNT5α, and E-cad in endometrial cancer (EC) and their clinical significance. The expression of UBE2C, WNT5α, and ZEB1 in 125 cases EC tissues were detected by immunohistochemistry. Patients clinicopathological, demography, and follow-up data were also collected. Positive rates of expression of UBE2C and ZEB1 were significantly higher in EC tissues when compared with the control tissues. The positive expression of UBE2C and ZEB1 were positively associated with tumor stages, local lymph node metastasis, and International Federation of Gynecology and Obstetrics (FIGO) stages. The positive rate of expression of WNT5a was significantly lower in EC tissues when compared with the control tissues. And positive expression of E-cad was inversely related to tumor stages, lymph node metastasis stages, and FIGO stages. Kaplan–Meier analyses demonstrated that positive expression of UBE2C or ZEB1 for EC patients had unfavorably overall survival time when compared with patients with negative expression of UBE2C or ZEB1. And EC patients with positive expression of WNT5a had favorably overall survival time when compared with EC patients with negative expression of WNT5a. Multivariate analysis demonstrated that positive expression UBE2C, WNT5α, and ZEB1, as well as FIGO stages were independent prognostic factors for EC patients. UBE2C, ZEB1, and WNT5a should be considered promising biomarkers for EC patients’ prognosis.

## 1. Introduction

Corpus uteri cancer is the second most commonly diagnosed cancer in female reproductive system worldwide.^[1]^ It is an estimated 417 thousand new cancer cases and 97 thousand deaths in 2020.^[1]^ Endometrial cancer is the most type of corpus uteri cancers. Estrogen is still considered to be a key factor in tumorigenesis and endometrial cancer (EC) cell proliferation, because in the absence of progesterone antagonism, continuous estrogen stimulation will drive the abnormal proliferation of endometrial epithelial cells.^[2]^ However, in some patients with subtypes that are not dependent on estrogen, the occurrence of tumors cannot be explained by abnormal levels of estrogen or progesterone. In addition, these subtypes are usually more malignant and resistant to various endocrine therapies.^[3]^ Tumor metastasis and recurrence are the main causes of failure of tumor treatment.

Ubiquitin-conjugating enzyme E2C (UBE2C), a ubiquitinating enzyme, could catalyze the degradation of proteins into smaller peptides, amino acids and ubiquitin in the 26S proteasome.^[4,5]^ It should be involved in a series of biological processes, such as cell cycle, apoptosis, and transcriptional process.^[6]^ Overexpression of UBE2C can promote tumorigenesis and be associated with unfavorable overall survival (OS) and progression-free survival of cancer patients.^[5,7–9]^

The wingless MMTV integration site (WNT) multigene family encodes secreted extracellular signaling proteins that regulate cell polarity, motility, and pattern during embryogenesis and tissue homeostasis. The WNT family contains19 highly conserved glycoproteins. Wingless MMTV integration site 5a (WNT5a) which is located on 3p14-p21 is a key member of WNT family and contains 5 exons. WNT5a is a key ligand for a single non-canonical WNT/β-catenin-dependent pathway.^[10]^ However, in some cases, it also activates a single WNT/β-catenin dependent pathway.^[11]^ WNT5a could promote activation of ERK1/2 in tumor cells. WNT5a plays an important role in cell adhesion, migration, as soon as in tumor development.^[12,13]^ Accumulating evidence have showed tant low expression of WNT5a should be associated with an unfavorable prognosis in many cancers.^[10,13,14]^

Zinc finger E-box binding homeobox1 (ZEB1) which can induce epithelial -mesenchymal transition (EMT) process through suppression Epithelial-cadherin (E-cad) expression is a driver of EMT and tumor progression.^[15,16]^ ZEB1 could regulate cell differentiation.^[17]^ Aberrant expression of ZEB1 has been found in many types of cancers, such as NSCLC, colorectal cancer, gastric cancer, EC, and pancreatic cancer.^[18,19]^

The purpose of this study is to detect the expression of UBE2C, WNT5a, and ZEB1 in EC tissues and assess correlations that they are associated with metastasis and prognosis in EC.

## 2. Methods

### 2.1. Patients and specimens

All 125 EC patients who were diagnosed at Department of Pathology of the First Affiliated Hospital of Bengbu Medical College, from January 2014 to December 2017. And 125 corresponding normal endometrial tissues from patients were used as the control group. Patients who received any chemo-radiotherapy were excluded. The study was approved by Bengbu Medical University ethical committee and performed in accordance with the guidelines issued of the Declaration of Helsinki. All specimens were obtained with patients written consent. The clinicopathological parameters, demography, and follow-up data of patients were collected. OS time was calculated from patients’ surgery date to her death date or December 2021. Clinical stages were evaluated by the basis of 2018 edition of the guidelines issued by International Federation of Gynecology and Obstetrics (FIGO) stages. Specific characteristics see Table 1.

**Table 1:** Patients characteristics.

Patient characteristics	Frequency (n)	Percentage (%)
Age (years)		
≤50	84	67.2
>50	41	32.8
Smoking		
No	101	80.8
Yes	24	19.2
Alcohol		
No	48	38.4
Yes	77	61.6
Size (cm)		
≤2.0	44	35.2
>2.0, ≤5.0	70	56.0
>5.0	11	8.8
Type		
Endometrioid	72	57.6
Mucinous	36	28.8
Serous	12	9.6
Clear cell	5	4.0
ER expression		
Negative	58	46.4
Positive	67	53.6
PR expression		
ER	77	61.6
PR	48	38.4
T stages		
T1	43	34.4
T2	68	54.4
T3	14	11.2
N stages		
N0	69	55.2
N1	43	34.4
N2	13	10.4
FIGO stages		
Ⅰ	26	20.8
Ⅱ	72	57.6
Ⅲ	27	21.6

FIGO = Federation of Gynecology and Obstetrics.

### 2.2. Immunohistochemistry

All samples (both EC group and control group) were fixed in 10% buffered formalin solution and embedded in paraffin, then cut into 4 μm thickening slices, and were deparaffinized and dehydrated with xylene and graded alcohol. Subsequently, all slices were rinsed with phosphate buffer solution (PBS, pH 7.2) for 10 minutes, then used methanol containing 3% hydrogen peroxide solution to block endogenous peroxidase activity of tissues and put into 95℃ citrate buffer solution (pH 6.0) for 30 minutes for antigen repair. Rinsed with PBS several times, and then were used goat serum for block for 20 minutes. Lastly, incubated with rabbit polyclonal antibody against human UBE2C (1:1000, rabbit monoclonal, ab252940; Abcam, UK) WNT5a (1:200, rabbit polyclonal, ab235966, Abcam), and ZEB1 (1:100, rabbit monoclonal, ab87280, Abcam) at 4℃ overnight. Replacing the primary antibodies by PBS, negative control staining was using performed, the slides were treated with polymer enhancer (Reagent A) for 20 min at room temperature. After a complete wash in PBS, the slides were treated with goat anti-mouse antibody (Reagent B) for 30 min at room temperature. After a complete wash in PBS, the slides were develop in freshly prepared diaminobenzedine solution for 8 minutes, and then counterstained with hematoxylin, dehydrated, airdried, and mounted. Immunohistochemical staining method was conducted in accordance with the Elivision^TM^ Plus (Maixin Biotechnology Co., Ltd, Fujian Province, China) detection kit instructions.

### 2.3. Evaluation of staining

Immunohistochemical staining results were evaluated by 2 experienced pathologists who were blind to patient data independently analyzed immunostaining results. Firstly, randomly selected 10 high-power field fields of every slice. Immunostaining results were evaluated accordingly to the product of staining extent and intensity.^[20]^ The intensity of positivity was scored as follows: 0, negative; 1, weak; 2, moderate; 3, strong. The extent of positivity was scored according to the percentage of cells showing positive staining: <10% is 1; 11% to 50% is 2; 51% to 75% is 3; >75% is 4. The final score was determined by multiplying the intensity of positivity and the extent of positivity scores, yielding a range from 0 to 12. For EC tissues that were positive expression of UBE2C, WNT5a, and ZEB1, mean score from each biomarker was calculated in tumor cells (The positive expression for biomarkers was scored as follows: >2, UBE2C; >2, WNT5a; and >2, ZEB1). When score > 2 was considered positive.

### 2.4. Statistical analysis

Relationships between clinicopathological characteristics and UBE2C, WNT5a, and ZEB1 expression were used Chi-square test or Fisher’s exact test. Associations among UBE2C, WNT5a, and ZEB1 expression were used Spearman’s test. The effects of UBE2C, WNT5a, and ZEB1 expression on OS were defined using Kaplan–Meier method with Log-rank test for univariate analysis. Multivariate analysis was used COX regression model. SPSS 19.0 software (SPSS 19.0 software, Chicago) for Window was used statistical analysis. When *P* < .05 was considered statistically significant.

## 3. Results

### 3.1. UBE2C, WNT5a, and ZEB1 expression in EC, and their associations with clinicopathological parameters

**Figure 1.**
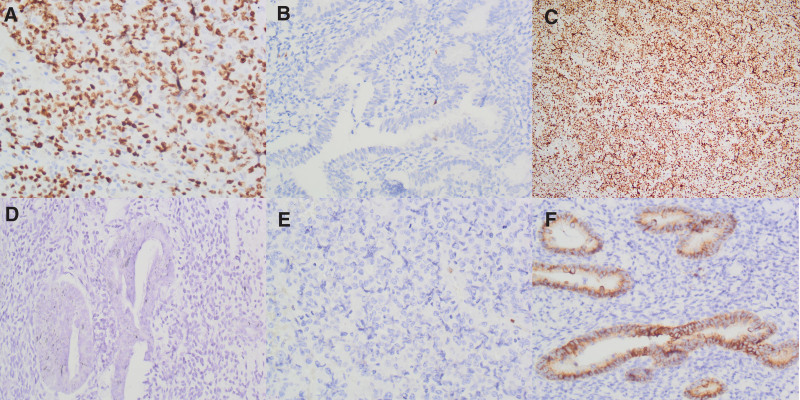
Immunostaining of UBE2C or ZEB1 or Wnt5a in EC or the control tissues. A: Positive staining of UBE2C in nuclei of the EC cells (400 magnification); B: Negative staining of UBE2C in the control tissues (400); C: Positive staining of ZEB1 in the nuclei of the EC cells (200); D: Negative staining of ZEB1 in the control cells (400). E: Negative staining of Wnt5a in the EC cells (400); F: Positive staining of Wnt5a in the cytoplasm of the control cells (400). EC = endometrial cancer, UBE2C = ubiquitin-conjugating enzyme E2C, WNT5a = wingless MMTV integration site 5a, ZEB1 = Zinc finger E-box binding homeobox1.

The positive immunostaining of UBE2C and ZEB1 were located in both nucleus of EC tissues, that of WNT5a was located in the cytoplasm. Overall, positive expression of UBE2C was 57.6% (72/125) in EC tissues and 9.6% (12/125) in the control tissues (see Fig. 1A and B). There was a significant difference between the 2 groups (*P* < .001). Expression of UBE2C was significantly related to tumor stages, lymph node metastasis (LNM), and FIGO stages, but not to patients age, smoking and alcohol status, gross type, and tumor diameter (see Table 2).

**Table 2:** The associations between expression of UBE2C, ZEB1, and Wnt5a and clinicopathological characteristics of EC.

Variables	UBE2C		*P*	ZEB1		*P*	Wnt5a		*P*
	−	+		−	+		−	+	
Age (yr)									
≤50	35	49	.812	34	50	.676	42	42	.522
>50	18	23		15	26		23	18	
Smoking									
No	44	57	.589	42	59	.263	52	49	.813
Yes	9	15		7	17		13	11	
Alcohol									
No	17	31	.212	18	30	.759	28	20	.263
Yes	36	41		31	46		37	40	
Type									
Endometrioid	43	29	<.001	37	35	.006	25	47	<.001
Mucinous	7	29		6	30		25	11	
Serous	1	11		4	8		10	2	
Clear cell	2	3		2	3		5	0	
Size (cm)									
≤2.0	30	14	<.001	21	23	.352	16	28	.025
>2.0, ≤5.0	21	49		24	46		41	29	
>5.0	2	9		4	7		8	3	
ER expression									
Negative	18	40	.017	16	42	.013	38	20	.005
Positive	35	32		33	34		27	40	
PR expression									
Negative	24	53	.001	24	53	.020	45	32	.068
Positive	29	19		25	23		20	28	
T stage									
T1	29	14	<.001	20	23	.479	16	27	.036
T2	21	47		24	44		39	29	
T3	3	11		5	9		10	4	
N stage									
N0	38	31	.006	37	32	<.001	29	40	.027
N1	12	31		12	31		26	17	
N2	3	10		0	13		10	3	
FIGO stages									
Ⅰ	21	5	<.001	17	9	.002	7	19	.003
Ⅱ	26	46		27	45		38	34	
Ⅲ	6	21		5	22		20	7	

EC = endometrial cancer, FIGO = Federation of Gynecology and Obstetrics, UBE2C = ubiquitin-conjugating enzyme E2C, WNT5a = wingless MMTV integration site 5a, ZEB1 = Zinc finger E-box binding homeobox1.

There was a significant difference between ZEB1 expression in EC tissues (60.8%, 72/125) and in the control group 6.4% (8/125; *P* < .001; see Fig. 1C and D). Expression of ZEB1 was significantly associated with tumor diameter, tumor stages, LNM, and FIGO stages, but not with patients age, smoking and alcohol status, gross type, and tumor location (see Table 2).

WNT5a expression is 88.8% (111/125) in the control group and 48.0% (60/125) in the tumor group (see Fig. 1E and F). The difference of WNT5a expression was significant between the 2 groups (*P* < .001). WNT5a expression was negatively correlated with tumor stages, LNM, and FIGO stages, but not with patients age, smoking and alcohol status, gross type, tumor location, and tumor diameter (see Table 2).

### 3.2. Univariate and multivariate analyses

**Figure 2.**
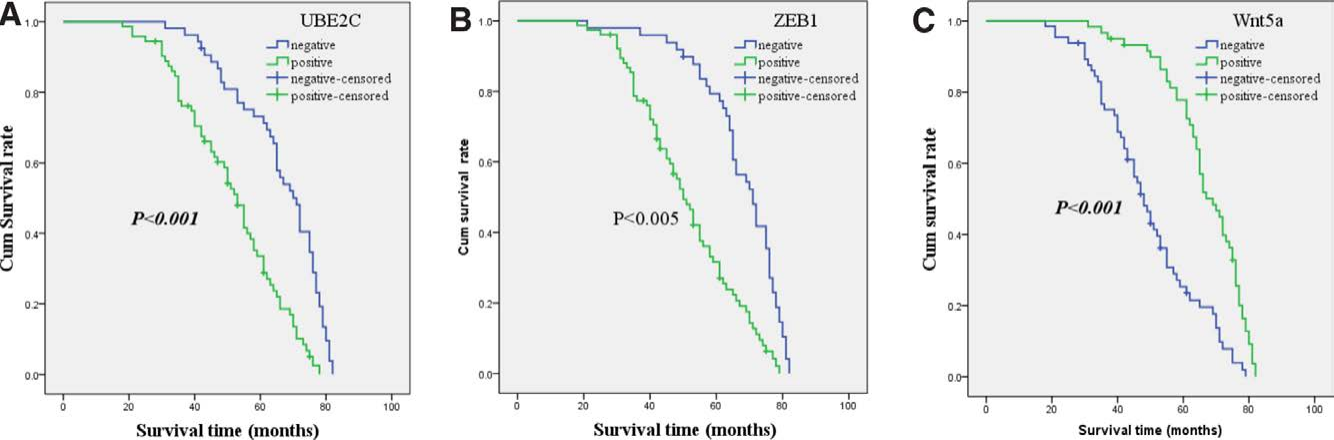
Kaplan–Meier analysis of the survival rate of patients with EC. The y-axis represents the percentage of patients; the x-axis represents their survival in months. A: Overall survival (OS) of all patients in relation to UBE2C (log-rank = 28.069, *P* < .001); B: OS of all patients in relation to ZEB1 expression (log-rank = 29.902, *P* < .001); C: OS of all patients in relation to Wnt5a (log-rank = 33.955, *P* < .001). In A–C analyses, the green line represents patients with positive expression of biomarkers and the blue line representing the negative expression of biomarkers. EC = endometrial cancer, UBE2C = ubiquitin-conjugating enzyme E2C, WNT5a = wingless MMTV integration site 5a, ZEB1 = Zinc finger E-box binding homeobox1.

As seen in Figure 2A, the survival curve indicated that OS time of EC patients who expressed UBE2C was more unfavorable than that of patients who not expressed the protein (65.6 ± 14.0 vs 49.9 ± 15.2 month, log-rank = 28.069, *P* < .001). Similar to UBE2C, the survival curve demonstrated that OS time of patients who expressed ZEB1 was also more unfavorable than that of patients who not expressed the protein (67.3 ± 12.9 vs 49.6 ± 15.0 month, log-rank = 29.902, *P* < .001; see Fig. 2B). Inversely to UBE2C, the survival curve suggested that patients who expressed WNT5a was more favorable than that of patients not expressed WNT5a (48.0 ± 14.9 vs 65.8 ± 13.0 month, log-rank = 33.955, *P* < .001; seen Fig. 2C).

Multivariate analysis demonstrated that positive expression of UBE2C, ZEB1, and WNT5a, FIGO stages were independent prognostic indicators for EC patients (see Table 3).

**Table 3:** Correlation among expression of UBE2C, ZEB1, and Wnt5a in EC.

Variable	UBE2C		*r*	*P*	ZEB1		*r*	*P*
	−	+			−	+		
UBE2C								
−					35	18	0.472	<.001*
+					14	58		
Wnt5a								
−	12	53	−0.504	.001†	10	55	−0.508	<.001†
+	41	19			39	21		

EC = endometrial cancer, UBE2C = ubiquitin-conjugating enzyme E2C, WNT5a = wingless MMTV integration site 5a, ZEB1 = Zinc finger E-box binding homeobox1.

* Positive association.

† Negative association.

### 3.3. Associations among the expression of UBE2C, ZEB1, and WNT5a in EC

The spearman correlation coefficient analysis suggested a negative association between WNT5a expression and UBE2C (*r* = −0.504, *P* < .001) or ZEB1 (*r* = −0.508, *P* < .001) expression. There was a positive association between UBE2C expression and ZEB1 expression (*r* = 0.472, *P* < .001) (see Table 4).

**Table 4:** Results of multivariate analyses of overall survival (OS) time.

Covariate	B	SE	*P*	HR	95% CI
FIGO stages	0.682	0.295	.021	1.978	1.109–3.528
UBE2C	0.609	0.274	.026	1.839	1.074–3.150
ZEB1	0.537	0.243	.027	1.711	1.062–2.758
Wnt5a	−0.614	0.258	.017	0.541	0.327–0.897

FIGO = Federation of Gynecology and Obstetrics, UBE2C = ubiquitin-conjugating enzyme E2C, WNT5a = wingless MMTV integration site 5a, ZEB1 = Zinc finger E-box binding homeobox1.

## 4. Discussion

Endometrial cancer is the second most common malignant tumor of the female reproductive system. The main reason of treatment failure of EC is recurrence and metastasis. In this study, through investigating in EC tissues and the control tissues of patients, we found that UBE2C and ZEB1 expression were high in EC tissues, while WNT5a expression was low in EC tissues. There were significant differences between EC tissues and the control tissues.

UBE2C protein is a ubiquitin-related enzymes that decide chain topology and catalyze the degradation of target proteins.^[8,21]^ The normal of this function is associated with completion of cell cycle and signal transduction.^[9,22]^ In our study, the result demonstrated that UBE2C expression is significantly higher in EC tissues than that in the control tissues. We found that UBE2C expression is positively rated to tumor stages, LNM, and FIGO stages. Kaplan–Meier analysis indicated that patients with positive expression of UBE2C had a shorter OS time when compared with patients who did not express UBE2C. The above results demonstrated that overexpression of UBE2C should play an important role in EC progression and metastasis and be considered as an important biomarker for prediction of EC patients’ prognosis.

ZEB1 which can induce EMT process through inhibition of E-cad activity is a critical inducer of EMT.^[16,23]^ ZEB1 plays a key role in regulation of cell differentiation and tissue specificity.^[17,19]^ In this study, the positive expression of ZEB1 in EC tissues is significantly higher than that in the control tissues. Furthermore, the positive expression of ZEB1 is significantly correlated to tumor stage, LNM, and FIGO stages. Kaplan–Meier analysis showed that patients with positive expression of ZEB1 had a more unfavorable OS time when compared with patients who did not express ZEB1. These results also indicated that overexpression of ZEB1 should participate in the process of progression and metastasis of EC, and also should be considered as a useful biomarker for prediction of EC patients’ prognosis.

WNT/β-catenin signaling pathway can regulate cell growth, proliferation, apoptosis and migration, which are closely related to the invasion and metastasis of various malignant tumors.^[12,13]^ WNT5a is an important member of WNT family. WNT5a can inhibit WNT/β-catenin signaling pathway by bind to tyrosine kinase, and activate WNT/β-catenin signaling pathway by interaction with Frizzled and low-density lipoprotein receptor -related transmembrane proteins.^[24]^ In this study, WNT5a expression is higher in the control group than that in the EC group. We found that WNT5a expression is negatively associated with tumor stage, LNM, as well as FIGO stages. Kaplan–Meier analysis indicated that patients with positive expression of WNT5a had a favorable OS time when compared with patients with negative expression of WNT5a. The above results demonstrated that low- or loss-expression of WNT5a should promote EC progression and metastasis, and also be considered as a promising biomarker for EC patients’ prognosis.

The previous study had demonstrated that WNT5a could inhibit EMT through inhibition Twist and ZEB1, thus suppressing the tumor mobility and invasion.^[10,25]^ WNT5a can also inhibit the canonical WNT signal pathway by weakening β-catenin. Abnormal expression of WNT5a should lose its ability of inhibition of movement and invasion of EC.^[14]^ UBE2C knockdown can inhibit tumor cells proliferation, migration, invasion, and EMT,^[12,13]^ therefore, overexpression of UBE2C should promote tumor cells invasion and EMT.^[8]^ However, the methodology of subjects in our study was relatively simple; further studies with more methodologies (such as in vitro and in vivo models) are needed to verify the present observation.

## 5. Conclusions

This study demonstrated that expression of UBE2C, ZEB1, and Wnt5a were associated with duration of OS among patients with EC. So, UBE2C, ZEB1, and Wnt5a should be considered as useful and valuable biomarkers in EC patients for prediction of invasiveness and prognosis.

## Acknowledgments

We thank all staffs at the Department of Pathology of our hospital for assistance with data and project management.

## Author contributions

**Conceptualization:** Yan Zhang.

Funding acquisition: Yan Zhang.

Investigation: Yingying Gong.

Methodology: Xueting Li, Zenong Cheng.

Resources: Yan Zhang, Danli Du.

Software: Huilei Chen.

Supervision: Lei Liu.

Writing – original draft: Yan Zhang.

Writing – review & editing: Yan Zhang.
